# REPLICCAR II Study: Data quality audit in the Paulista Cardiovascular Surgery Registry

**DOI:** 10.1371/journal.pone.0223343

**Published:** 2020-07-10

**Authors:** Bianca Maria Maglia Orlandi, Omar Asdrúbal Vilca Mejia, Gabrielle Barbosa Borgomoni, Maxim Goncharov, Kenji Nakahara Rocha, Lucas Bassolli, Pedro Gabriel Melo de Barros e Silva, Marcelo Arruda Nakazone, Alexandre Sousa, Valquíria Pelisser Campagnucci, Karlos Alexandre de Sousa Vilarinho, Marcelo Katz, Marcos Gradim Tiveron, Magaly Arrais dos Santos, Luiz Augusto Ferreira Lisboa, Luis Alberto de Oliveira Dallan, Fábio Biscegli Jatene, REPLICCAR II Study Group

**Affiliations:** 1 Department of Cardiovascular Surgery, Instituto do Coração do Hospital das Clínicas da Faculdade de Medicina do Estado de São Paulo (INCOR), São Paulo, São Paulo, Brazil; 2 Department of Cardiovascular Surgery, Hospital Samaritano Paulista, São Paulo, São Paulo, Brazil; 3 Department of Cardiovascular Surgery, Hospital De Base de São José do Rio Preto, São José de Rio Preto, São Paulo, Brazil; 4 Department of Cardiovascular Surgery, Beneficência Portuguesa de São Paulo, São Paulo, São Paulo, Brazil; 5 Department of Cardiovascular Surgery, Irmandade da Santa Casa de Misericórdia de São Paulo, São Paulo, São Paulo, Brazil; 6 Department of Cardiovascular Surgery, Hospital das Clínicas da Universidade Estadual de Campinas, Campinas, São Paulo, Brazil; 7 Department of Cardiovascular Surgery, Hospital Israelita Albert Einsten, São Paulo, São Paulo, Brazil; 8 Department of Cardiovascular Surgery, Irmandade da Santa Casa de Misericórdia de Marília, Marília, São Paulo, Brazil; 9 Department of Cardiovascular Surgery, Instituto Dante Pazzanese de Cardiologia, São Paulo, São Paulo, Brazil; Universidade de Mogi das Cruzes, BRAZIL

## Abstract

The quality of data in electronic healthcare databases is a critical component when used for research and health practice. The aim of the present study was to assess the data quality in the Paulista Cardiovascular Surgery Registry II (REPLICCAR II) using two different audit methods, direct and indirect. The REPLICCAR II database contains data from 9 hospitals in São Paulo State with over 700 variables for 2229 surgical patients. The data collection was performed in REDCap platform using trained data managers to abstract information. We directly audited a random sample (n = 107) of the data collected after 6 months and indirectly audited the entire sample after 1 year of data collection. The indirect audit was performed using the data management tools in REDCap platform. We computed a modified Aggregate Data Quality Score (ADQ) previously reported by Salati et al. (2015). The agreement between data elements was good for categorical data (Cohen κ = 0.7, 95%CI = 0.59–0.83). For continuous data, the intraclass coefficient (ICC) for only 2 out of 15 continuous variables had an ICC < 0.9. In the indirect audit, 77% of the selected variables (n = 23) had a good ADQ score for completeness and accuracy. Data entry in the REPLICCAR II database proved to be satisfactory and showed competence and reliable data for research in cardiovascular surgery in Brazil.

## Introduction

The very foundation of healthcare and clinical studies, as well as clinical trials and follow-up studies, is the quality of the data collected. Despite the lack of consensus regarding a standardized method to measure healthcare data quality, it is of utmost importance to establish the confidence and validity of the outcome. Hence, the research design, the variable selection and the data collection are pivotal points in asserting the accuracy of the conclusion achieved [1].

Observational studies are subject to bias, confounding and a lack of information in retrospective medical records. Publications such as Zhang et al., 2014, Salati et al., 2015, and Dreyer et al., 2016, were important initiatives on how to devise data validation tools aimed at enhancing the quality of the results needed for decision-making [2–5]. It is essencial that healthcare databases are reliable, as they will not only be used as the basis for future academic research, but also to evaluate and derive guidelines leading to the improvement of healthcare decision-making [6,7–10].

For decades, cardiac surgeons have systematically collected and analyzed data to continually improve outcomes in healthcare [10]. The initiatives taken by the Society of Thoracic Surgeons (STS) and European Society of Thoracic Surgeons (ESTS) are aimed at collection of reliable data on a large scale in order to improve outcomes, especially regarding mortality. Adhering to a quality improvement initiative with data registries can already reduce mortality rates [11–20].

In the 2010 audit report for the STS Adult Cardiac Surgery Database (ACD), Brown and colleagues [21] emphasize the importance of data quality, which had previously not been taken into consideration. That same year, Grukemeier and Furnary [22] also addressed this curiously neglected problem. One initiative is the Paulista Registry for Cardiovascular Surgery (REPLICCAR II).

The the REPLICCAR II registry is a voluntary initiative with 9 participating centers located in Sao Paulo (Brazil) and is coordinated by the Instituto do Coração do Hospital das Clínicas da Faculdade de Medicina da USP (InCor-HCFMUSP). The main objective is to evaluate morbidity and mortality predictors in patients undergoing Coronary Artery Bypass Graft Surgery (CABG). The adoption of quality-oriented data analysis was the next step taken in REPLICCAR II to assure the validity of outcomes and enhance its clinical impact.

The aim of the present study is to present direct and indirect audit results of data quality in the REPLICCAR II database, after 6 months and 1 year.

## Material and methods

Ethics and Consent form of this work was approved as a subproject of the Ethics Commission for Research Project Analysis (FAPESP) of HCFMUSP, under online registry number 2016/15163-0, entitled “Ampliação e Aprimoramento do Registro Paulista de Cirurgia Cardiovascular através de parceria com o Registro do Estado de Massachusetts/Harvard University para melhoria da qualidade dos Programas em Cirurgia Cardíaca no Sistema Único de Saúde”.

### Data source and collection

The project included 9 institutions in the State of São Paulo, thus combining the analysis of public and private reference hospitals linked to institutions such as philanthropic organizations and universities. Funding was provided by Fundação de Amparo à Pesquisa do Estado de São Paulo (FAPESP).

Data collection and management used REDCap (Research Electronic Data Capture) hosted at University of São Paulo Medical School General Hospital (HCFMUSP) accessible from any computer with an Internet connection, with access restricted to selected researchers [23,24]. The criteria and definitions in REPLICCAR II were exactly the same as in the STS ACD (version 2.9, 2017) and includes more than 700 variables, such as general characteristics, risk factors, pre-, intra-, and postoperative assessments and their 30-day follow-up.

Data collection began in August 2017 and each participating center mobilized a task force headed by a supervisor, usually a medical resident. The participating centers received training and a codebook with the description and criteria of all study variables to collect from medical records after isolated CABG surgery.

Thirty variables (27 mandatory fileds and 3 additional variables) were selected for the direct and indirect data quality analysis. These variables were selected based on the principal variables reported in CABG surgery studies. Variables related the samples caracteristics, pre, intra and postoperative were collected. The complications are a combination of clinical outcomes after surgery, such as: renal failure, reoperation, atrial fibrillation, deep sternal wound infection, stroke, myocardial infarction, respiratory complications, prolonged use of mechanical ventilation, etc.

### Direct audit

A direct audit was conducted 6 months after the initiation of data collection. Seven percent (n = 107 records) of the medical records for each surgical patient at each center through February 2018 were randomly selected with STATA 13.1 software (StataCorp, Texas, USA). Data were re-collected by two experienced auditors and 2 data managers (who also collect data routinally). Having full access to each center’s data, the audit was performed under two conditions: (i) that data managers were blinded to the original record and (ii) each data manager would not re-collect the same cases they had originally input. The original and the re-collected data then underwent statistical analysis to check for accuracy in data collection.

### Indirect audit

In the second phase of the study, a direct audit was impracticable due to the amount of data and the lack of financial and human resources. The 30 variables used in direct audit were used to compare data collected between 6 months and 12 months.

We evaluated all records inputed in REDCap (indirect audit) using the data management tools available in the platform [23,24]. To ensure the completeness of data elements in the REPLICCAR, all electronic data collection forms were programmed to alert the data manager of failure to fill a field (variable). Numeric fields were validated to prevent the entry of non-numeric characters or numbers outside an acceptable range. Futhermore, REDCap has a module for assessing the quality of recorded data. This module has standard tools that quickly describe all missing values; fields with invalid characters; outliers values and incorrect values for calculated fields. In addition to the REDCap data quality standard tools, specific queries were defined to identify inconsistent values. The customization of specific queries for the REPLICCAR enabled construction of measures to screen for data inconsistencies. Table 1 shows some examples of REPLICCAR queries.

**Table 1:** REPLICCAR queries examples. REPLICCAR II, 2019.

Variable / Study time	Querie description
**Pre-surgery**	
Renal failure and Serum creatinine	([dialysis] = 1 and [renal_failure] = 0) or ([renal_failure] = 1 and ([creatinine]< 1,2 mg/dl and [creatinine_clearence]≥ 90 ml/min))
Diabetes, blood glucose and Hemoglobin A1c	([diabetes] = 0 and [HbA1c] > 8,0) or ([diabetes] = 0 and [glucose] ≥ 200) or ([diabetes] = 1 and [diabetes_treatment] = 0)
**Surgery**	
Blood transfusion and red blood cell count	([transfusion] = 1 and ([lowest_hemoglobin]>12 and [lowest_hematocrit] > 50))
**Post-surgery**	
LVEF variation > 50%	([postop_lvef]–[preop_lvef]) ≥ ([preop_lvef]/2) or ([preop_lvef]–[postop_lvef]) ≤ (([preop_lvef]/2)*(-1))
Complications and hospitalization time	([postop_complications] = 1 and ([hospitalization_days] > 30 or [postop_days] > 15))

### Statistical analysis

For the direct audit, the data was analyzed in STATA v.13.1 (StataCorp, Texas, USA).

To evaluate agreement of categorical variables, Cohen’s *kappa* coefficient (κ) was applied. *Kappa coefficient* (κ) was reported as (10): (i) fair, when between 0.21 and 0.4; (ii) moderate, when between 0.41 and 0.6; (iii) substantial, when between 0.61 and 0.8; and (iv) almost perfect, when between 0.81 and 1 (8). The Intraclass Correlation Coefficient (ICC) was determined for continuous variables (2-way Random-Effects Model for reliability of agreement). The ICC varies between 0 and 1, with the former suggesting no agreement, and the latter suggesting perfect agreement. Values lower than 0.5 are indicative of poor reliability. Those between 0.5 and 0.75 were moderate, those between 0.75 and 0.9 good, and those higher than 0.9 were of excellent reliability [9].

For the indirect audit, we adapted the methodology suggested by Salati et al, implemented by the REPLICAR II responsible team, by assessing completeness (COM) of data, and the accuracy (ACC) of inconsistent or out of range answers for all data included in the study (n = 2229 medical registries).

In this fashion, as follows [4]:

**Completeness (COM)** = (1 − (‘null values’/total expected values)) × 100

**Accuracy (ACC) =** [1− (‘inconsistent values’/ total expected values)] × 100

**Rescaled COM** = COM of the Unit − (average COM of all the examined Units/standard deviation of all the examined Units)

**Rescaled ACC** = ACC of the Unit − (average ACC of all the examined Units/standard deviation of all the examined Units)

**Aggregate Data Quality Score (ADQ) = Rescaled COM + Rescaled ACC**

The ADQ score illustrates the both completeness and accuracy of variables in the study.

Negative ADQ score indicates that the observed ACC/COM is inferior to the sample average, whereas positive values show that the sample average is superior to the observed ACC/COM.

## Results

### Direct audit

A total of 107 random records for direct data audit analysis were collected in the initial 6 months of REPLICCAR II Study (7% of the total sample) and are summarized in Table 2. The sample average kappa (κ) was 0.70, with standard error of 0.06 (95%CI 0.59–0.83). We observed in this analysis mostly substantial kappa’s coefficient (n = 4) to almost perfect (n = 3), with 2 variables presenting a moderate κ coefficient.

**Table 2:** Direct audit: Inter-rater agreement and estimated κ coefficient of categorical variables. REPLICCAR II, 2019.

Variables	Inter-Rater Agreement (%)	Estimated κ (SE)
Family history of coronary heart disease	91.7	0.62 (0.10)
*Diabetes mellitus*	96.3	0.93 (0.09)
*Diabetes* treatment	87.3	0.76 (0.11)
Dyslipidemia	88.8	0.78 (0.09)
Renal failure	92.1	0.42 (0.09)
Dialysis	100	1.00 (0.10)
Hypertension	97.3	0.86 (0.09)
Intra operative blood transfusion	91.7	0.75 (0.10)
Post op complications	75.0	0.47 (0.09)
Myocardial Infarction	95.2	0.64 (0.2)
Atrial Fibrilation	81.0	0.62 (0.2)
Renal Failure	90.5	0.61 (0.2)

Table 3 presents the ICC of numerical variables collected in the study in the direct audit. Preoperative hemoglobin had an average ICC = 0.7, but it later became clear that there were many different admissions in laboratory examinations, leading to data disagreement about this situation between data managers (inter rater agreement).

**Table 3:** Direct audit: ICC of numerical variables with, two-way random effects model. REPLICCAR II, 2019.

Variables	ICC	95% CI
	(Average)	
**Pre-operative**		
Age	0.86	0.80–0.91
Height (cm)	0.98	0.96–0.98
Weight (kg)	0.99	0.98–0.99
Hemoglobin (mg/dL)	0.70	-0.65–0.96
Glucose (mg/dL)	0.99	0.98–1.00
Ejection fraction (%)	0.99	0.98–0.99
**Intra-operative**		
Lowest intraop hematocrit (%)	0.98	0.96–0.99
Highest intraop glucose (mg/dL)	0.97	0.96–0.98
Intraop perfusion time (min)	0.99	0.98–0.99
Intraop anoxia time (min)	0.99	0.996–0.998
**Post-operative**		
Ejection fraction (%)	0.94	0.80–0.98
Glucose (mg/dL)	1.00	0.99–1.00
Hematocrit (%)	0.98	0.95–0.99

Data for glycated hemoglobin, total bilirubin, and albumin levels were insufficient for the analysis, but these variables were not mandatory in the registry. The mandatory variables for the direct audit had on the overall satisfatory results with agreement up to 90%, demonstrating a good adherence of data managers to the study definitions and criteria.

### Indirect audit

Table 4 describes the completeness and accuracy for all variables included in the current evaluation and the ADQ score. The variables with less than 90% of completeness (COM) and low ADQ score in preoperative period were: (i) total bilirubin (14%), (ii) total albumin (21.8%), (iii) *HbA1c* (41.7%), (iv) glucose (60.5%), and (v) ejection fraction (75.13%). In the postoperative period, there were only two variables in this condition: (i) ejection fraction (22.3%) and (ii) glucose (73.9%).

**Table 4:** ADQ score of completeness and accuracy of REPLICCAR II database (n = 2229), 2019.

Variables	COM (%)	Rescaled COM	ACC (%)	Rescaled ACC	ADQ
**Pre Operative**					
Age (years)	97.2	0.54	99.8	-0.50	0.03
BMI (kg/cm2)	96.7	0.51	98.5	-5.29	-4.78
Family history CHD	97.9	0.56	100	0.23	0.80
*Diabetes mellitus*	98.1	0.57	100	0.23	0.80
*Diabetes* treatment	95.9	0.48	100	0.23	0.72
Dyslipidemia	97.4	0.54	100	0.23	0.78
Renal failure	97.8	0.56	100	0.23	0.79
Dialysis	97.9	0.56	100	0.23	0.80
Hypertension	97.9	0.56	100	0.23	0.80
Rheumatic disease	96.3	0.50	100	0.23	0.73
Hemoglobin (mg/dL)	92.7	0.35	100	0.23	0.59
Hematocrit (%)	92.5	0.34	100	0.23	0.58
Total albumin (g/L)	21.8	-2.51	100	0.23	-2.28
Total bilirubin (mg/dL)	14.0	-2.82	100	0.23	-2.59
Glucose (mg/dL)	60.5	-0.95	100	0.23	-0.71
HbA1c	41.7	-1.70	100	0.23	-1.47
Ejection fraction (%)	75.1	-0.36	100	0.23	-0.12
Creatinine (mg/dL)	92.9	0.36	100	0.23	0.59
**Intra Operative**					
Lowest intraoperative hemoglobin	96.9	0.52	100	0.23	0.75
Lowest intraop hematocrit	96.9	0.52	100	0.23	0.75
Highest intraop glucose	96.6	0.51	100	0.23	0.74
Intra op blood transfusion	95.9	0.48	100	0.23	0.72
**Post Operative**					
Ejection fraction (%)	22.3	-2.49	100	0.23	-2.25
Glucose (mg/dL)	73.9	-0.41	100	0.23	-0.17
Creatinine (mg/dL)	94.9	0.44	100	0.23	0.68
Hemoglobin (mg/dL)	90.0	0.24	100	0.23	0.48
Hematocrit (%)	90.0	0.24	100	0.23	0.48
Post op complications	95.7	0.47	100	0.23	0.71
Post op duration^†^	95.3	0.46	100	0.23	0.69
Hospitalization total (days)^†^	95.3	0.46	99.94	-0.01	0.44
Mortality (OUTCOME)	94.9	0.44	99.81	-0.50	-0.06

† Calculated fields: admission and discharged dates input in the platform.

The ADQ score for body mass index (BMI) was -4.7, because weight and height inconsistences. The values outside the expected range were notificated to the responsible data manager within query to revise the data (Table 4). The remaining variables presented more than 90% completeness and accuracy. The variable of ejection fraction was absent in the hospital database and was not included by the data managers. However, this could account for exams given directly to a surgeon and lost in the REPLICCAR II database.

**Fig 1:**
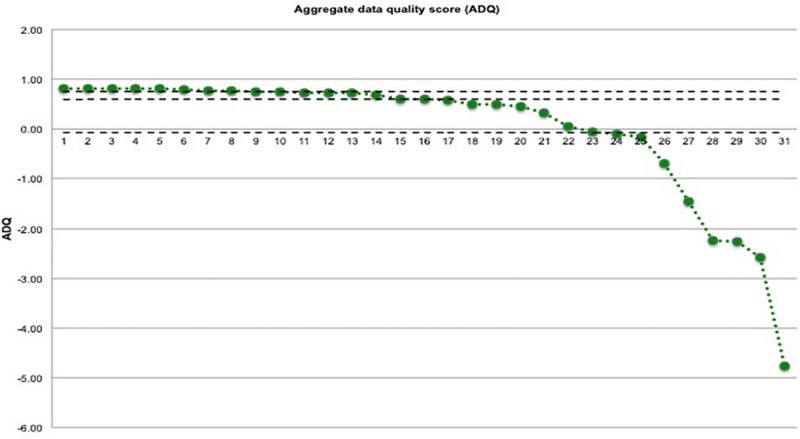
ADQ score for the variables analyzed in the indirect audit. REPLICCAR II, 2019.

Fig 1 shows that 77% of the records (n = 23) had an acceptable ADQ score, considering that the positive values had a larger ADQ score than the sample average, which can be considered of good data quality. The values under the first quartile were considered relevant for review.

Considering this interpretation, we propose better criteria and definitions (Table 5) for some variables in the REDCap tool, including the BMI, with ranges for weight and height. During data input, investigators received an alert for each piece of data determined to be out of range, thus guaranteeing improvement of accuracy, data consistency and acceptable variability (queries).

**Table 5:** Rules applied to the RedCAP tools after the indirect audit considering better criteria and definitions to improve data quality in REPLICCAR II Study. REPLICCAR II, 2019.

Field Label	Calculations	Text Validation			Branching Logic
		Type	Min	Max	
**Age**	round (datediff([dob], [surgdt], "y", "dmy"))	number			
**Gender**	1, Male | 2, Female				
**Admission date**		date_dmy	01/08/17		
**Height (cm)**		number	120	250	
**Weight (Kg)**		number	30	300	
**BMI**	[weightkg]*10000/([heightcm]*[heightcm])				
**Family History CHD**	1, Yes | 2, No				
**Diabetes mellitus**	1, Yes | 2, No | 3, Unknown				
**Diabetes Control**	0, No control | 1, Diet | 2, Oral | 3, Insulin | 4, Others subcutaneous | 5, Others not subcutaneous | 6, Unknown	autocomplete			[diabetes] = '1'
**Dislypidemia**	1, Yes | 2, No | 3, Unknown				
**Renal Failure**	1, Cronic | 2, Acute | 3, No | 4, Unknown				
**Dialysis**	1, Yes | 2, No				
**Hypertension**	1, Yes | 2, No | 3, Unknown				
**Rheumatic Disease**	1, Yes | 2, No | 3, Unknown				
**Hemoglobin (mg/dL)**		number	5	20	
**Hematocrit (%)**		number	15	70	
**Last creatinin (mg/dL)**		number	0.1	30	
**Creatinin clearance**	round (if([cirurgia_arm_1][gender] = 1, (140-[cirurgia_arm_1][age])* [cirurgia_arm_1][weight]/(72* [cirurgia_arm_1][creatlst]), if([cirurgia_arm_1][gender] = 2, (140-[cirurgia_arm_1][age])*[cirurgia_arm_1][weight]/(72*[cirurgia_arm_1][creatlst])*0.85,NaN),”2”)	number			
**Total Albumin (g/L)**		number	1	10	
**Total Bilirrubin (mg/dL)**		number	0.1	10	
**Glucose (mg/dL)**		number	20	500	
**HbA1c**		number	1	20	
**Lowest Intraop Hemaglobin**		number	1	50	
**Lowest Intraop Hematocrit (%)**		number	1	99.99	
**Highest Intraop Glucose (mg/dL)**		number	40	500	
**ICU stay (hours)**	sum([icuduration],[icuadhrs],"h","dmy",true)	number			[icuvisit] = '1'
**Mortality**	1, Yes | 2, No				[icuvisit] = 0
**Date of death**		date_dmy			[mtopd] = 1
**Survival (days)**	datediff([surgdt],[mtdate], "d", "dmy")	number			
**Hospitalization total (days)**	datediff([admitdt],[dischdt],"d","dmy",true)	number			
**Post Op duration (days)**	datediff([surgdt],[dischdt],"d","dmy",true)	number			
**Date of Birth**		date_dmy			
**Surgery Date**		date_dmy			
**Hospital Discharge Date**		date_dmy			
**Date of Death**					[mtopd] = 1

BMI: Body Mass Index; CHD: coronary heart disease; creatlst: last creatinin; ICU: Intensive Care Unit; icuvist: entrance in ICU after surgery; dob: date of birth; admitdt: admission date; surgdt: surgery date; dischdt: discharge date; mtdate: date of death; dmy: date/month/year.

The other variables with low completeness are not mandatory but reflect our reality and highlight this opportunity to improve clinical evidence and quality protocols.

## Discussion

In summary, the REPLICCAR II study had satisfactory concordance in the first stage, and the results of the indirect analysis were essential to develop methods of data confidence and quality improvement.

Lauricella et al, 2018 [7], published a data quality analysis on a similar initiative developing such a database. The São Paulo Lung Cancer Registry (PLCR), also developed by InCor, cannot be directly compared with REPLICCAR II, due to its different parameters. However, it is possible to analyze and compare some of these parameters, such as COM. With 511 analyzed records, 21 out of 105 variables (20%) had COM < 0.9%. In our study, 7 out of 30 variables (23.3%) showed the same results. The work by Salati et al, 2011 [1], showed that 5 out of 15 variables (33.3%) selected for the study had < 90% completeness.

In analyzing direct audit results, it’s important to remember that ICC considers that close numerical values might be concordant, even if they are different. This has important implications in a clinical study, because different values within close range will show good ICC values. Considering that different researchers (or even the auditor) may collect information, such as exam values, from different dates for the same subject, these values may show good ICC in the statistical analysis [23]. Only 2 out of 15 variables (13.3%) had ICCs inferior to 0.9. Our lowest value was 0.7 ICC for preoperative hemoglobin. Lauricella et al. (2018) reported equivalent results found in 5 out of 12 numerical variables (41.7%). Their lowest ICC value was 0.51 for the “time from first symptom” variable. The comparison, however, cannot be applied directly to our groups, due to the completely different parameters in each work.

Grunkemeier and Furnary (2010) have commented on the methodology for direct audit in the STS ACD, published by Brown et al. (2010), with the main objective of determining the variability of disease etiology and operative data elements in the STS ACD, when abstracted by untrained physician abstractors. Their discussion focused on which method was the most trustworthy in the direct audit, and argued that a more interesting analysis could be conducted by comparing the data managers with themselves [22]. In our study, the data managers evaluated other data managers to ensure comparable analysis.

According to Shahian et al. (2010): “Determining optimum clinical care and developing evidence-based guidelines require the highest quality study data. As we enter an era of greater transparency and accountability, data accuracy has even more widespread implications”. Direct audit increases costs and human resources required and in large databases such stragedy is not feasible. [25].

Regarding outcome variables in our study, mortality had 85% completeness and 92% accuracy in the first audit phase. Among the inconsistencies related to mortality, we verified that cases of intraoperative death were negligible for the variable death in the operating room. To rectify such inconsistency, we inserted in the REDCap platform queries that considers the cases of surgery without admission to intensive care unit at the immediate postoperative period as death on the day of surgery. The variables “Mortality in Hospital” or “30 Days Vital Status” had 96% completeness and 99.7% accuracy.

The STS (2008) reported similar results, where only 83% of cases had agreement related to patient status (alive or dead). Brown et al. (2010) reported a total agreement median of 93% (range 35–100%) in CABG procedures in the STS ACD. Patients with unknown or incomplete 30-day mortality status could potentially introduce bias into any analyses not adopting a statistically valid strategy to handle missing information. This is a concerning situation, considering that mortality is the most common outcome used for both quality indicators and research [21].

However, “in-hospital mortality” completeness was almost entirely recorded, representing the vast majority of 30-day deaths. The use of simulations suggests that any errors, committed by the unrealistic assumption that a patient with missing or unknown mortality status is alive, have negligible influence on hospital mortality results when compared with random sampling error. [15].

Newly proposed parameters, such as ADQ, may provide faster, more practical and lower-cost analysis of generic data quality. Another evaluation with ADQ score to evaluate quality between institutions, as Salati et al. (2015) made, could then be used to orient the centers about the strengths and weaknesses of their respective variables, thereby helping them to improve data quality.

The STS ACD study adapted a conceptual framework of quality measurement with a comprehensive methodology for quality assessment, which strive every day to continually improve it, like a process of evolution and not a static product [25].

### Limitations

1. Our work was limited due to financial and human resources. Thus, a direct audit or a more restricted follow-up of the centers was impracticable. Nonetheless, faced with such difficulty, our team is looking forward for new perspectives in data quality analysis, such as ADQ, thus contributing to the development of the area.
2. As expected from such a pioneering project, there were many challenges regarding the education of the professionals engaged in the collection and ensuring data quality, as shown by the unexpected discrepancies in our results. Considering solely the analysis made (κ coefficient and ICC), we cannot understand the causes of these errors. In addition, it was not possible to confirm if all cases meeting the criteria (primary procedure and isolated CABG) were included, we were unable to properly evaluate the centers’ adherence.
3. We have found satisfactory concordance, but these results only show the capacity and understanding of the investigators to collect data in the first phase of study (6 months after beginning). Considering that most centers rely on medical residents for the data collection, we cannot ensure long-term adherence of each center because it is expected that a short to medium term rotation of these professionals will occur with the training having to be restarted. Consequently, the continuous quality analysis is imperative to keep COM/ACC (preferably above 98%).
4. This work shows that there is still a long way to go before we can develop a Brazilian national database comparable to the STS or the ESTS databases. It is still not possible to ensure that professionals, researchers, healthcare centers, and the government will adhere to the promotion and adoption of electronic registries long term. Nonetheless the development of a consensus for a broad database is growing.
5. Another limitation is related to the 30 days follow up. We haven’t made an audit about the outcomes in this period or re-call patients to confirme data.

In summary, this work shows seriousness and commitment to REPLICCAR project, being concerned not only with the study development and implantation, but also with the quality of its data.

## Conclusion

Completeness and accuracy of the information abstracted from medical records are essential to the validity and accuracy of the results obtained. Indirect auditing gave clear directions for data improvement, without the need to recollect a sample to evaluate concordance.

Above all, it is important to maintain a scientific partnership between institutions with regular meetings, thereby closely integrating with working groups in each institution. Findings of discrepancy within the data only reinforce the need for quality-oriented statistical studies, because it directly influences validity, analysis and conclusions performed in research. In places where such studies and their application are still underdeveloped like Brazil, studies in this field become even more indispensable. Focusing on data quality is a sure factor that ultimately leads to a more efficient and safer healthcare system and will play an increasingly major role in its development. The main objective of the present study was to implement improvement actions in such a way that guarantees safety and validity to the results, as well as to provide feedback on REPLICCAR II itself. As an STS-based database, this project can provide the basis for a wider and more reliable quality-oriented program, with the prospect of a positive impact on clinical outcomes.

Our experience reinforces the importance of training, encouraging and standardizing the staff responsible for collecting and filling out the forms (data managers). In addition, correctly entering data substantially lowers the costs of direct audit with the traditional Raters Agreement Analysis. Indirect auditing was more practical in determining strategies for data quality improvement, but direct audit was essencial for evaluate outcomes definitions and improve education and training. ADQ scores consider the completeness and accuracy of each variable in the study and show the best data quality parameters in prospective observational studies. It is therefore expected that it will attract more attention in studies yet to come.
